# Finding the Right Questions: Exploratory Pathway Analysis to Enhance Biological Discovery in Large Datasets

**DOI:** 10.1371/journal.pbio.1000472

**Published:** 2010-08-31

**Authors:** Thomas Kelder, Bruce R. Conklin, Chris T. Evelo, Alexander R. Pico

**Affiliations:** 1 Department of Bioinformatics – BiGCaT, Maastricht University, Maastricht, The Netherlands; 2 Gladstone Institute of Cardiovascular Disease, San Francisco, California, United States of America; University of California Davis, United States of America

## Abstract

This Essay discusses the role of pathways for exploratory data analysis in present-day biology.

At a time when biological data are increasingly digital and thus amenable to computationally driven statistical analysis, it is easy to lose sight of the important role of data exploration. Succinctly defined over 30 years ago by John Tukey [1],[2], exploratory data analysis is an approach to data analysis that focuses on finding the right question, rather than the right answer. In contrast to confirmatory analysis, which involves testing preconceived hypothesis, exploratory data analysis involves a broad investigation, a key component of which may be visual display. Though his arguments predate personal computing and thus focus on graph paper and ink, the point still stands: good data visualization leads to simpler (better) descriptions and underlying fundamental concepts. Today, there is tremendous potential for computational biologists, bioinformaticians, and related software developers to shape and direct scientific discovery by designing data visualization tools that facilitate exploratory analysis and fuel the cycle of ideas and experiments that gets refined into well-formed hypotheses, robust analyses, and confident results.

## Pathways for Exploratory Data Analysis

**Figure 1:**
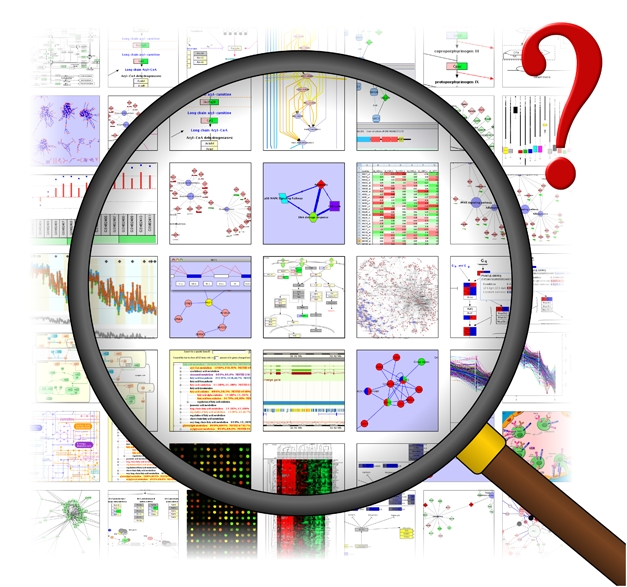
Pathways for exploratory data analysis. Biological pathways are powerful visualization tools for data exploration, focused on finding the right question.

A rich source of visual material relevant to the study of biology is pathway diagrams. Pathways map our understanding about connections and processes underlying biological function. They are powerful models for exploring, interpreting, and analyzing biological datasets and provide a medium to apply Tukey's exploratory data analysis principles to the present-day study of biology (Figure 1). Pathways organize and visualize data and provide a model that both computers and humans can work with, since they are abstract enough to allow for semi-automatic integration and querying in a biological context, and biologists are by and large familiar with pathway diagrams. Ongoing efforts to capture biological knowledge in pathway databases [3] and data exchange formats [4] demonstrate growing interest in applying pathway visualization and analysis to biology research.

Currently, several bioinformatics tools provide pathway visualization to support the exploration of datasets [5],[6]. DeRisi et al. projected the changes in mRNA expression on the carbon and energy metabolism pathway to create a visual representation of the properties of metabolic reprogramming during the diauxic shift of yeast [7]. Bensellam et al. applied similar visualization techniques to connect beta cell physiology to specific metabolic and signaling pathways in rat islet cells [8]. A pathway also incorporates a collection or set of biological entities (e.g., genes, proteins, metabolites) that function in the biological process described by the pathway. This information can be used to reduce the dimensionality of large datasets. Identifying pathways that are overrepresented with entities showing interesting behavior gives an overview of global patterns among different biological processes. Many tools and techniques implement this principle [6],[9], and it has become an integral part of gene expression data analysis [10]. Recent innovations utilize connectivity and weighting in the calculation of pathway impact [11]. These techniques produce a list of putatively affected pathways that serves as a basis for researchers to develop testable hypotheses of mechanism or direct further exploration. Importantly, when pathway representations are employed in exploratory data analysis, the goal is not a statistical solution, but rather an investigation of the scope of the data and relevant patterns. Pathways serve as the medium for communication, in which the biological story is extracted from the data, prior knowledge is integrated and understanding is constructed [12].

## Challenge

An important goal of “-omics” experiments is to generate directed hypotheses based on relatively noisy but large-scale datasets, which can then be tested in targeted experiments. In this respect, exploratory and confirmatory approaches are complementary, where applying exploratory techniques is a logical first step in the analysis [2]. The relationship is actually more iterative than sequential, where a certain level of statistical analysis or reduction might be required before applying an exploratory technique. But in the overall trajectory from exploratory to confirmatory, exploration is most important in forming a conclusive statistical approach. In the field of pathway analysis, there is active research in developing new techniques and tools from the confirmatory paradigm, using pathways to improve statistical power on specific hypotheses [9],[11],[13]–[16]. The value of these techniques for exploratory analysis, however, is limited in the absence of a comprehensive framework for exploration and visualization. The challenge we face now is to fill this gap and to develop flexible tools and pathway content based on the exploratory data analysis paradigm.

Looking at hallmarks of exploratory data analysis may suggest ways that pathways can be more effectively used in data exploration. We will discuss three properties that typify both the exploratory technique and analyst: flexibility, interactivity, and effectiveness. By relating properties of exploratory data analysis to the current state of pathway analysis techniques, we hope to guide researchers in how to best utilize pathway information in exploratory data analysis and help focus future tool development towards better exploratory pathway analysis techniques.

## Flexibility

Exploratory analysis is not a linear start to end process with fixed analysis steps but requires flexibility from both researchers and tools. The decision on what will be the next step in an exploratory analysis is guided by the data and observations rather than by a predefined plan, as is the choice for the technique that is most suitable for highlighting the features under investigation. In exploratory data analysis, we look at the data from many different points of view, few of which actually lead to new or relevant observations. But knowing that a certain description of the data does not lead to a new or relevant observation is itself a step forward in the analysis. The following analogy from Tukey illustrates this:

“As detective stories remind us, many of the circumstances surrounding a crime are accidental or misleading. Equally, many indications to be discerned in bodies of data are accidental or misleading. To accept all appearances as conclusive would be destructively foolish, either in crime detection or in data analysis. To fail to collect all appearances because some—or even most—are only accidents would, however, be gross misfeasance…” [1].

Thus, open-mindedness is important when using pathways for exploratory data analysis and provides software developers with both a challenge and an opportunity. It is hard to create versatile software that does not restrict researchers to a single workflow. A more generic, flexible framework to support various pathway analysis procedures would be very powerful and would provide a basis for developing new and better pathway analysis techniques. Therefore, instead of aiming for a single, isolated software package, developers should implement flexible solutions that can be integrated in a larger toolbox for pathway analysis, in which each tool provides a different perspective on the dataset. In turn, rather than depending on a single program or algorithm to produce a publishable statistic, biologists should seek tools that help comprehend the data, view it from different angles, and thereby lead to greater understanding of what's going on.

Consider canonical pathways. These pathways summarize complex biological processes in a comprehensible way, however, these summaries may omit important details by grouping entities, leaving out alternative routes, and imposing artificial boundaries. By limiting analysis to canonical pathways, a researcher is less flexible, fixated on well-described knowledge, and blind to less certain, but potentially more interesting clues. Reality is much more complex than what is depicted in the typical canonical pathway, as has been demonstrated by available protein–protein interaction networks [17] and curated interaction databases, such as Reactome [18]. However, visualizing every possible interaction or entity that might contribute to a process can lead to large incomprehensible “hairball” networks that do not facilitate exploratory analysis. How can we optimally use both types of information in an exploratory analysis?

One option might be to consider canonical pathways as a starting point in the analysis, based on solid foundations from which we might explore less known but potentially interesting areas. For example, a pathway could be dynamically extended with interactions from other pathways, protein–protein interactions, or relations from literature, based on a set of entities that show interesting behavior in the dataset under investigation. In that way, the researcher can explore instances or interactions that might not be integral to the canonical pathway, but might still be relevant to the observations in the pathway. This process could become data-driven, by highlighting and filtering information that is potentially interesting based on the experimental data and context, instead of showing all available information. An analysis environment that exploits both canonical pathways and detailed interaction networks would encourage researchers to take a flexible, exploratory attitude and facilitate construction of an understandable biological story from complex data.

For developers, realizing that exploratory pathway analysis tools might be used not only in isolation but also with other software and different types of data in a flexible analysis setup might guide software design and implementation. For example, providing an application programming interface (API) in addition to the user interface greatly enhances the flexibility to adapt a tool for customized analyses or to reuse components. Reusability of software components that perform common tasks and define general data models leads to more unity among pathway analysis tools. For example, a data format will be more easily adopted by other developers when an API is available to read, modify and write it. In addition, providing an API opens up the possibility for scripting to automate tasks and combine functionalities of different tools. This introduces a nearly unlimited flexibility and allows a developer to focus on the main functions of a tool and keep the user interface simple and focused, while keeping the option open for advanced users to automate and combine standard features of different tools to perform a novel type of analysis.

## Interactivity

An exploratory analysis is not an automatic process, but relies on decisions by the researcher. Where calculation or visualization tasks may fall to the computer, the researcher controls interpretation and decisions on what data should be viewed, from which angle and in which context. Graphical representations of data are important. As Tukey notes, a good visualization “forces us to notice what we never expected to see,” and “The graph paper (or visualization software) is there, not as a technique, but rather as recognition that the picture-examining eye is the best finder we have of the wholly unanticipated” [2]. Interactive graphics allow the researcher to take control of how the data are visualized and stimulates the researcher to change the visualization perspective based on previous observations.

Pathway analysis techniques that allow the researcher to explore data interactively (rather than delivering a static view) will facilitate exploration and increase the chance of finding interesting observations or patterns. There are several opportunities to improve interactivity of pathway visualizations and highlight features relevant to the question being asked while, just as importantly, filtering out irrelevant features.

Geographical maps illustrate the advantages of interactivity provided by effective visualization software. Paper maps divide the world into multiple views of fixed scope and scale. You can look at a map of the complete world with limited detail or a city map without context. But paper maps are cumbersome and lack critical interactivity (folding a map doesn't count). Digital maps, on the other hand, have several advantages, such as the ability to switch scale through interactive zooming, so you can scroll the viewport to trace a possible route or track your real-time location with GPS information. The integration of information, in general, is yet another advantage, as you can add and remove layers of information on the same map. Such integrated information can be interactively queried to find a particular intersection, a high concentration of public parks, or the best route through traffic. The parallels to biological pathways are obvious and should be exploited at every opportunity in the design of pathway analysis tools. The example of traffic overlays even hints at the dynamics of biological processes, e.g., the flow of biochemistry through metabolic pathways.

Developers of exploratory pathway analysis tools could borrow concepts from the analogy with geographical maps. For example, enrichment analysis techniques group genes, proteins, and metabolites at the level of pathways ranked by activity. This provides a global “world map” view, showing which pathways may be affected while discarding information about the inner workings of these pathways. This scale may hold information on how each pathway acts as a unit in a specific context and how these units relate to each other. Such relationships could include child–parent relations (glucose metabolism and fatty acid metabolism are both metabolic pathways), the flow of substances (the output of glycolysis is an input for the TCA cycle) or causal relations (the P53 pathway regulates apoptosis). In contrast to the global scale, techniques based on the constituents of pathways provide a more mechanistic “city map” view by relating data to localized interactions and reactions. Continuing to zoom to the molecular level reveals protein domains, the exon structure of splice variants, and polymorphisms. Interactivity may be improved by allowing seamless transitions between these scales by utilizing semantic zooming [19], where the displayed features and level of detail change automatically along with the zoom level and context. Given that most analysis tools focus on pathway information at a single scale, switching between these scales within an exploratory analysis is far from trivial.

## Effectiveness

The interactive, user-directed character of exploratory data analysis imposes stricter criteria on the effectiveness of exploratory techniques. The techniques described in Tukey's textbook on exploratory data analysis are surprisingly simple and easy to apply merely with paper and pencil. This allows the researcher to take a quick look at typical questions—“could it be that…?” or “what if it is the case that…?”—without investing days of work on that single question. Effective techniques that are relatively easy to apply and work in a transparent way encourage the researcher to take a true exploratory attitude instead of following well-trod paths while ignoring side roads that may reveal unexpected but interesting aspects of the data.

Of course, if the chance of finding an interesting observation in the data does not outweigh the efforts to perform an analysis technique, researchers may decide not to use the technique. This problem may be less relevant in confirmatory approaches, where investing a large effort in a single technique is often justified because the effort versus results can be weighed during planning. However, in exploratory analysis, a single technique is only a small part of the whole analysis (many clues need to be considered, with different techniques), and the yield is often unpredictable (many clues lead to dead ends). Therefore, the acceptable maximum effort is very low, and to make pathway analysis techniques suitable for true exploratory analysis, this should be taken into account.

Unfortunately, many obstacles and annoyances exist when applying current pathway analysis techniques. While modern computers allow fast data processing and visualization, there remain numerous hurdles beyond the need to install and train on multiple software packages and the need to format and reformat datasets into specific input formats. Reordering data columns might not be a major hurdle—spreadsheet software that performs this task is widely available. But mapping data to different identifier systems or applying calculations on the data is less trivial and more prone to error, often requiring specific bioinformatics skills. Pathway analysis tools should aim to remove the responsibility of data reformatting from the researcher by making tools more flexible to different types of input data or to adhere to widely adopted standards. Generic libraries and services that might assist the developer in this task are already available, such as BridgeDb [20] for identifier mapping (to support multiple identifier systems), Web services to access the latest pathway information [21]–[24], or paxtools [25] for reading pathways in the BioPAX standard.

The pathways themselves require library-like organization and curation. A handful of projects have undertaken the task of capturing and curating this knowledge as semantic content that is amenable to computation [18],[21],[26]–[28]. Unlike systems biology networks, pathways cannot be directly inferred from high-throughput data, but rather require the synthesis of multiple discoveries, insights, and diverse data types spanning years, or even decades, of work by multiple groups, offering an opportunity for tool developers to facilitate the entry, curation, and distribution of pathway content in effective formats [4],[28],[29]. BioPAX and SBGN are particular examples of community-driven formats for pathway semantics and graphical notation, respectively. Pathways should be understandable by researchers who may not be fully familiar with the biological process that is described, enabling researchers to look at data in context of knowledge outside the scope of their specialty [5]. The most effective pathways are self-explanatory, contain detailed information about biological context, and reference relevant primary data sources and literature.

Another opportunity to make exploratory pathway analysis techniques more effective is to work on better integration with public data resources. Biologists create a wealth of data, which is often available in a public repository, such as ArrayExpress or GEO for transcriptomics datasets [30],[31]. During an exploratory analysis, it can be valuable to extend beyond the researcher's own data to consider relevant orthogonal or correlated datasets. However, this is an inefficient process. The researcher must manually find the right datasets, download the data files from the repository, reformat the data, and import it in the pathway analysis tool. An increasing number of public repositories support Web service queries, assisting developers in building tools that perform these tasks programmatically [32]. Repositories and tools that expose data and methods through Web services can readily be integrated into effective, reusable workflows in pathway analysis tools, leading to high-order standards in data analysis.

Effective data integration is a significant hurdle in working with different datasets and pathways in exploratory analysis. Determining what to integrate and how to present it to the user depends on the context and the question being asked. However, this context is typically defined at the semantic level and, thus, is hard for computers to work with. For example, a computer can easily handle the command “hide everything above a certain *p*-value threshold,” but has trouble with “show me all data related to cancer.” In an ideal situation, the data are annotated with this information, but the computer still needs to deal with synonyms or subtypes of the word “cancer.” It becomes even more complex when integrating data at the pathway level, where the researcher could ask something like “show me all studies in which MYC is activated by MAPK.” Such questions require correctly annotated pathway information and must deal with information at the semantic level (which interactions “activate”) and synonym or identifier mapping problems (which entities map to “MAPK”).

Recent developments begin to address these issues. Ontologies help in dealing with information at the semantic level. For example, a disease ontology could tell the computer that melanoma is a subtype of cancer, and a event ontology could tell the computer that activation could include phosphorylation, translation or receptor binding interactions. Standards for ontologies, such as the OBO format, and resources that provide access to different ontologies through unified Web services [33] provide the necessary interfaces for tool developers to improve integration of different types of data in pathway analysis tools. In addition, data repositories are actively working on annotating raw datasets to provide better context [34],[35], ready to be queried by pathway analysis tools through Web interfaces. Sometimes referred to as integromics, or multi-omics, the integration of annotations and data is critical to extracting the full potential from large and high-throughput datasets [9],[36],[37]. Effective construction, analysis and visualization of multi-omic datasets depend on innovative software. These tools must understand what is going in (i.e., with the help of ontologies and data exchange standards), know how to merge and normalize across orthogonal data types, and be adept at displaying multi-dimensional information in meaningful and intuitive contexts. This is a particularly ripe area for exploratory tool developers.

## Conclusion

Biological pathways are a powerful medium in the exploratory analysis of biological datasets, providing a conceptual framework that is familiar to biologists, visually oriented and increasingly available in digital formats that allow interactive display and analysis. By discussing properties of exploratory data analysis in the light of pathways, we highlighted several opportunities for researchers and developers to use pathway analysis in an exploratory setting. Rather than trying to provide a complete overview of pathway analysis approaches, we discussed several ideas and recent developments that lay out a path towards a powerful set of pathway analysis tools developed from an exploratory analysis paradigm. A critical recurring issue is that current pathway analysis tools are rather isolated and hard to combine within an analysis. This may discourage researchers to follow clues that require the use of a different tool to view the data from another perspective, thereby standing in the way of a true exploratory attitude. The field of exploratory pathway analysis is still in its beginning, but with focused and coordinated development, it may eventually play an important role in providing the right questions for confirmatory approaches.
